# Oral mucosal adverse events with chlorhexidine 2 % mouthwash in ICU

**DOI:** 10.1007/s00134-016-4217-7

**Published:** 2016-02-05

**Authors:** Nienke L. Plantinga, Bastiaan H. J. Wittekamp, Kris Leleu, Pieter Depuydt, Anne-Marie Van den Abeele, Christian Brun-Buisson, Marc J. M. Bonten

**Affiliations:** 1 Julius Center for Health Sciences and Primary Care, University Medical Center Utrecht, P.O. Box 85500, 3508 GA Utrecht, The Netherlands; 2 Department of Intensive Care, AZ St. Lucas Hospital, Groenebriel 1, 9000 Ghent, Belgium; 3 Department of Intensive Care, Ghent University Hospital, De Pintelaan 185, 9000 Ghent, Belgium; 4 Clinical Microbiology Laboratory, AZ St. Lucas Hospital, Groenebriel 1, 9000 Ghent, Belgium; 5 Medical ICU and Infection Control Unit, Hopitaux Universitaires Henri Mondor and Université Paris-Est Créteil, 94000 Créteil, France; 6 Department of Medical Microbiology, University Medical Center Utrecht, P.O. Box 85500, 3508 GA Utrecht, The Netherlands

Dear Editor,

Oral care using a chlorhexidine solution is commonly used as an infection prevention measure in European ICUs [1]. The preventive effects of different decontamination strategies, one of which is mouthwash with chlorhexidine digluconate 2 % (CHX 2 %), on the incidence of ICU-acquired bacteremia with multidrug-resistant bacteria is being investigated in a multicenter cluster-randomized study in 13 ICUs in six European countries [www.clinicaltrials.gov NCT02208154] (see Supplementary Material for detailed methods). An unexpected high incidence of oral mucosal lesions was observed in ICU patients receiving CHX 2 %.

Oral mucosal lesions, including erosive lesions, ulcerations, white/yellow plaque formation, and bleeding mucosa were observed in 29 of 295 patients (9.8 %) that had received CHX 2 % in the first two hospitals testing this intervention (Supplementary Table S1, Pictures 1–4). The median time to onset of oral lesions was 8.0 days (IQR 4.5–11.0) in the 24 patients in whom duration of exposure could be ascertained. CHX 2 % was discontinued prematurely in 16/29 cases and oral mucosal lesions disappeared after cessation of CHX 2 % in all patients.

Patient characteristics were comparable for the baseline (*n* = 310) and CHX 2 % periods (*n* = 295; Supplementary Table S2) for the two ICUs. During the baseline period CHX 0.20 and 0.12 % were used for oral care in hospitals A and B, respectively, without evidence of oral lesions in any patient. All other procedures related to oral care remained identical in each hospital during both periods.

Amongst the CHX 2 % treated patients, occurrence of side effects was associated with male gender, APACHE II score, length of stay in the ICU, and duration of mechanical ventilation, suggesting a dose-response relationship, with increasing risks of oral mucosal lesions for the more severely ill patients, undergoing mechanical ventilation, and receiving CHX 2 % for longer periods (Table 1). This hypothesis is supported by the localization of the lesions in the oral cavity; most lesions occurred where stasis of the mouthwash might have occurred—despite suctioning after administration—such as below the tongue and in the buccal pockets.

**Table 1:** Baseline characteristics of CHX 2 % treated patients with and without adverse events

	Adverse events (*N* = 29)	No adverse events (*N* = 266)	Pearson Chi square/indep. *t* test
Male gender	23 (79.3 %)	161 (60.5 %)	*P* = 0.047
Admission type			*P* = 0.155
Medical	13 (44.8 %)	153 (57.5 %)	
Trauma	5 (17.2 %)	20 (7.5 %)	
Surgical	11 (37.9 %)	93 (35.0 %)	
Acute illness (y/n)	24 (82.8 %)	198 (74.4 %)	*P* = 0.324
Antibiotic at ICU admission (y/n)	11/29 (37.9 %)	123/259 (47.5 %)	*P* = 0.328
Age, mean (SD)	60.4 (13.3)	60.1 (15.7)	*P* = 0.921
APACHE II, mean (SD)	26.7 (8.0)	19.6 (8.6)	*P* < 0.0005
ICU-LOS, median (IQR)	28 (21–41.5)	10.5 (6–19)	*P* < 0.0005 (LN)
Geometric mean (SD)	27.2 (1.8)	10.6 (2.2)	
Length of MV, median (IQR)	19 (14.5–28.5)	6 (3–11)	*P* < 0.0005 (LN)
Geometric mean (SD)	18.8 (1.8)	6.2 (2.3)	

*SD* standard deviation, *IQR* interquartile range,* LOS* length of stay, *LN* log-transformed variable, *MV* mechanical ventilation, *N* number of patients

Mechanical stress during application of CHX 2 % may have played a role in hospital A, where the solution was initially applied using Kocher’s forceps with gauzes and where the incidence seemed to have reduced after changing to application using a syringe. Hospital B had applied CHX 2 % with a gauze wrapped around a gloved finger.

In 12 patients symptoms predominantly consisted of pronounced white plaques at the tongue and other localizations in the mouth, in some resembling* Candida* infection (Supplementary Picture 3). Yet, the incidence rate ratio between prior respiratory tract colonization with *Candida* spp. (monitored twice weekly as part of the study protocol and in clinical cultures) and the occurrence of side effects was 0.94 (95 % confidence interval 0.09–1.79, Supplementary Table S3). An association with herpes reactivation could not be determined as reactivation was investigated in five affected patients only (Supplementary Table S1).

The study safety committee recommended to replace CHX 2 % mouthwash by a CHX 1 % oral gel in the remaining hospitals. Since then CHX 1 % was withdrawn for reasons of intolerance in 2 of 419 (0.5 %) patients in four hospitals, after 12 and 30 days of use.

On the basis of these findings, we recommend against the use of 2 % chlorhexidine digluconate mouthwash in ICU patients.

## Electronic supplementary material

Below is the link to the electronic supplementary material.

Supplementary material 1 (DOCX 192 kb)
