# Supplementary material for: Oral mucosal adverse events with chlorhexidine 2 % mouthwash in ICU
Source: Intensive Care Med. 2016 Feb 5;42(4):620–1. doi: 10.1007/s00134-016-4217-7 (PMC5413521; doi:10.1007/s00134-016-4217-7)
Supplement: Supplementary file 1 — Supplementary material 1 (DOCX 192 kb) [file 134_2016_4217_MOESM1_ESM.docx]

**Methods**

In this ongoing multicenter study, ICUs start with a baseline period of 6 months, during which oral care may include chlorhexidine 0.12% or 0.2% mouthwash when this is part of routine oral care. Subsequently, oral decontamination with CHX 2%, SOD or SDD is implemented in a randomized order per ICU, each intervention being applied for a 6-month period. Adult patients are eligible for inclusion if they have an expected length of mechanical ventilation of 24 hours or more. After inclusion, patients are treated according to the study protocol until extubation. Because of the ecological nature of the study and the perceived minimal risks associated with the interventions studied, a waiver for informed consent - or permission to perform the study with the possibility for patients or legal representatives to sign informed refusal - has been obtained from the local institutional review boards for all participating hospitals and, in countries where this was required, from the national regulatory authorities [1].

During the CHX intervention, 10 mL of a 2% chlorhexidine digluconate mouthwash solution was applied to the oral mucosa four times daily after performing standard oral care. Hospitals were instructed to administer CHX 2% by soaking a toothette (sponge) in the solution and gently applying it to the surface of the teeth, gumline and tongue. Excess chlorhexidine remaining in the oral cavity was to be removed by suctioning.

CHX 2% was produced according to GMP guidelines by the department of Clinical Pharmacy of the University Medical Center Utrecht (Sponsor of the study). The composition of the solution is shown in textbox S1 (supplement). Excipients used in the solution include sorbitolum liquidum cristallisabile, purified water, alcohol 96% v/v (7g/100ml) and Peppermint oil Ph. Eur. All these substances are also used in the production of the commercially available chlorhexidine digluconate 0.12% and 0.20% solutions (e.g. Corsodyl ®, Farmaclair, Hérouville St Clair, France), and no other substance was added to the solution.

Formal recording and reporting of adverse events was not part of the study protocol, but left to the discretion of investigators, unless such events met the definition of suspected unexpected serious adverse reactions (SUSAR). These are *serious* adverse events (events that result in either death, are life threatening, require hospitalization or prolongation of hospitalization and/or result in persistent or significant disability or incapacity) that are reasonably related to the intervention under study. The adverse events described here did not meet this definition and were therefore reported spontaneously by two participating sites and then analyzed.

Patient characteristics were compared between the baseline and CHX 2% periods and between patients with and without adverse events using Pearson’s Chi-square test for categorical variables and the two-sided independent samples t-test for continuous variables. Furthermore, associations between respiratory tract colonization with Candida species and adverse events were assessed.

*Reference*

1. Wittekamp BH, Wise MP, Brun-Buisson C, Bonten MJ, (2014) Regulatory obstacles affecting ecological studies in the ICU. The Lancet Infectious diseases 14: 913-915

**Textbox S1. Chlorhexidine 2% mouthwash formulation (p. 100 mL)**

|  | | | |
| --- | --- | --- | --- |
| Chlorhexidine digluconate solution 200 g/L Ph.Eur | 10.7 | gr | Active ingredient |
| Sorbitolum liquidum cristallisabile Ph.Eur. | 53.5 | gr | Taste adjuster |
| Peppermint oil Ph.Eur. | 150 | μl | Taste adjuster |
| Alcohol 96% v/v Ph.Eur. | 7 | gr | pH adjuster |
| Purified water Ph.Eur | 100 | ml | Solvent |

**Legend**

gr, gram; L, liter; ml, millilitre; Ph.Eur, European Pharmacopoeia (European quality standard); μl, microliter; v/v, volume per volume

**Table S1. Line-listing of patients who experienced adverse events during the use of CHX 2%**

|  | **Symptoms as described in medical file (remarks and consultations)** | **Days of onset adverse events ^a^** | **CHX stopped pre-maturely?** | **Candida ^b^** | **Herpes simplex ^c^** |
| --- | --- | --- | --- | --- | --- |
| **Hospital A** | | | | | |
| 1 | bleeding gums | 2 | yes | no | NT |
| 2 | yellow viscous plaque on whole lower lip, lesions in both cheeks at the height of the teeth, yellow +++, severe +++ | 1 | yes | no | NT |
| 3 | bleeding gums and white lesions in the entire mouth | 24 | yes | yes | NT |
| 4 | crustae lips, yellow lesion under the tongue, viscous plaque in throat, non-painful | 19 ^d^ | no | no | NT |
| 5 | diffuse white plaques in the pharynx and cheeks | 8 | yes | yes | NT |
| 6 | swelling of lips and mucosa, spontaneous bleeding lips, white plaque and starting white lesions of both cheeks at the height of the teeth | 4 | yes | no | NT |
| 7 | white tongue and 'atonic' colour of the gums | 10 | yes | no | NT |
| 8 | bleedy mouth (picture 3) | 6 | yes | no | NT |
| 9 | bleeding gums, one zone yellow plaque in mouth, white plaques on tongue | 8 | yes | yes | NT |
| 10 | bleeding gums, zones with yellow plaque in mouth | 2 | no | no | NT |
| 11 | yellow plaques with slight bleeding on inside of lips | 8 | no | no | NT |
| 12 | bleeding gums, no distinct ulcera, swelling mucosae and lips (pt with thrombocytopenia, 10*10^3^ / mm^3^) | 2 | no | yes | NT |
| 13 | tiny white bleeding ulcera near teeth pockets (picture 1) | 12 | yes | no | Positive  (oral lesion) |
| 14 | bleeding ulcerations gingival pockets ischemic lesions uvula palatum molle mucosal edema (consult stomatology: no necrotizing gingivitis) | 11 | yes | yes | Positive (nose) |
| 15 | bleeding gums near teeth pocket (consult stomatology: tiny ulcerations, no necrotizing gingivitis lesions caused by friction during mouth care) | 11 | no | no | Positive (ETA) |
| 16 | excessive mouth bleeding with clotting (consultation with ENT physician: no clear lesions in mouth. laryngoscopic examination by intensive care physician: palatum molle ulcerations matching endotracheal tube position lesions cauterized, bleeding stopped) | 10 | yes | yes | low positive (ETA) |
| 17 | bleeding ulcerations gingival pockets (consult stomatology: no necrotizing gingivitis) | 12 | no | no | low positive (ETA) |
| **Hospital B ^d^** | | | | | |
| 1 | dry tongue, "aphthous lesions", thickening (picture 2) | *unknown* | no | *unknown* | NT |
| 2 | white plaque | *unknown* | no | *unknown* | NT |
| 3 | white plaque | *unknown* | no | *unknown* | NT |
| 4 | yellow plaque | *unknown* | no | *unknown* | NT |
| 5 | white plaque (picture 4) | *unknown* | no | *unknown* | NT |
| 6 | white plaque tongue | 6 | no | no | NT |
| 7 | white plaque tongue | 8 | yes | no | NT |
| 8 | white plaque tongue, gums and lips | 11 | yes | yes | NT |
| 9 | "aphthous lesions" lower lip + gums | 14 | yes | yes | NT |
| 10 | white plaque tongue | 2 | no | no | NT |
| 11 | white plaques tongue and (open) "aphthous lesions" lower lip | 6 | yes | no | NT |
| 12 | white plaque tongue, open wound lower lip | 6 | yes | no | NT |

**Legend**ETA, endotracheal aspirate; NT, not tested.
^a^ Time between inclusion date and occurrence of adverse events.

^b^ Defined by the presence or absence of Candida spp. in any respiratory culture result (including oropharyngeal swabs), taken at least 1 day prior to the onset of adverse events.

^c^ Result from Herpes Simplex Q-PCR (body site), histology was not performed.

^d^ Adverse events occurred during the 2^nd^episode of mechanical ventilation, “days of onset” include 3 ventilator-free days.

^e^ The first 5 cases from hospital B were reported retrospectively, the date of onset of adverse events could therefore not be retrieved.

**Table S2. Baseline characteristics of patients included in the baseline period and the chlorhexidine 2% period (CHX 2%)**

|  | **Baseline period**  **(N=310)** | **CHX 2% period**  **(N=295)** | **Pearson Chi-Square / Indep. T-test** |
| --- | --- | --- | --- |
| Male gender | 192 (61.9%) | 184 (62.4%) | P = 0.912 |
| Admission type |  |  | P = 0.344 |
| Medical | 188 (60.6%) | 166 (56.3%) |  |
| Trauma | 18 (5.8%) | 25 (8.5%) |  |
| Surgical | 104 (33.5%) | 104 (35.3%) |  |
| Acute illness (y/n) | 251 (81.0%) | 222 (75.3%) | P = 0.089 |
| Antibiotic at ICU admission (y/n)  ^a^ | 55/144 (38.2%) | 134/288 (46.5%) | P = 0.100 |
| Age (SD) | 60.0 (15.0) | 60.1 (15.5) | P = 0.899 |
| APACHE II, mean (SD) | 21.6 (8.8) | 20.3 (8.8) | P = 0.056 |
| ICU-LOS, median (IQR)  geometric mean (SD) | 12 (6-22)  11.5 (2.4) | 12 (7-21)  11.6 (2.3) | P = 0.870 (LN) |
| Length of MV, median (IQR)  geometric mean (SD) | 6 (3-14)  6.9 (2.5) | 7 (3-13)  6.9 (2.4) | P = 0.966 (LN) |
| Candida-positive respiratory culture during ICU-admission | 143 (48.1%) | 120 (41.7%) | P = 0.115 |

**Legend**SD, standard deviation; IQR, interquartile range; LN, log-transformed variable; N, number of patients.
^a^ Differentially missing data; in one hospital, this variable was missing in 73.8% of baseline and 3.0% of CHX 2% patients.

**Table S3. Association between respiratory tract colonization with *Candida spp*. and the occurrence of adverse events**

|  | Patient days at risk (PD) | Adverse events (cases) | Incidence rate (1000 PD ^-1^) |
| --- | --- | --- | --- |
| Candida detected in respiratory tract | 879 | 8 | 9.10 |
| No candida detected in respiratory tract | 1651 | 16 | 9.69 |
|  | 2530 | 24 | 9.49 |

*Patients were considered at risk for adverse events from the start of CHX 2% until a) two days after extubation
or b) if prior to that date: until ICU-discharge. The first five cases from hospital B were excluded from this analysis, because the start date of adverse events was unknown.*


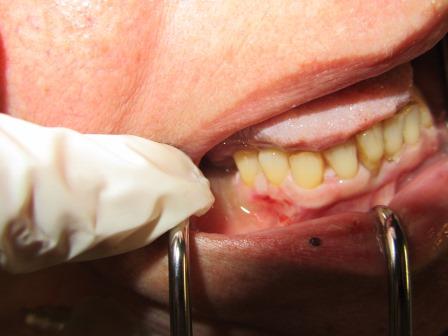
**Picture 1:** Case A13 with tiny white bleeding ulcera near teeth pockets …………

**Picture 2:** Case B1 with dry tongue, “aphthous lesions”, thickening


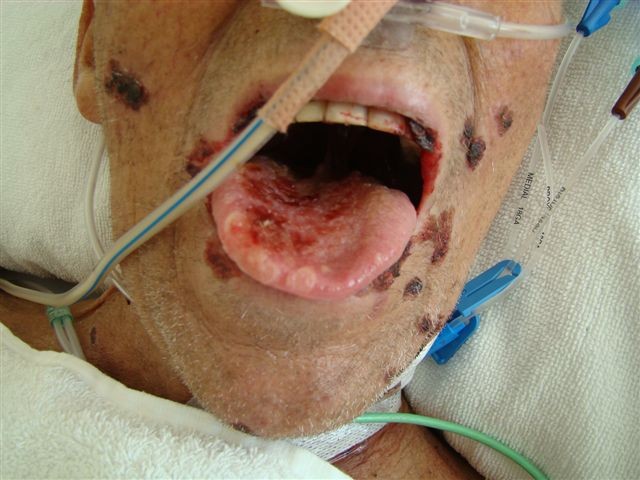

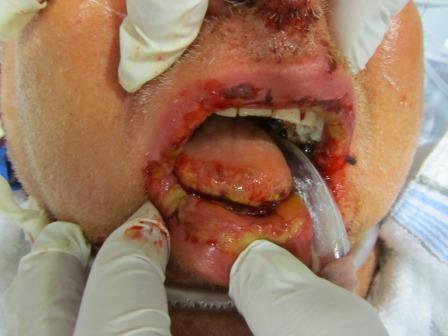


**Picture 3:** Case B5 with white plaque formation, visually resembling Candida stomatitis


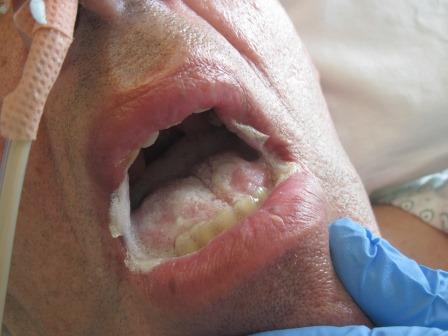


**Picture 4**: Case A8 with a bleeding mouth, visually resembling Herpes Simplex Virus reactivation
